# Diet Therapy and Probiotics to Improve Sleep Apnea Risk and Quality of Life in Older Adults (>60 Years) with Metabolic Syndrome: A Study from Romania

**DOI:** 10.3390/geriatrics10040100

**Published:** 2025-07-25

**Authors:** Amina Venter, Amin-Florin El-kharoubi, Mousa El-kharoubi, Evelin Claudia Ghitea, Marc Cristian Ghitea, Timea Claudia Ghitea, Ciprian Florian Venter

**Affiliations:** 1 Doctoral School of Biological and Biomedical Sciences, University of Oradea, 410087 Oradea, Romania; aminaadnan2005@yahoo.com; 2 Bihor Clinical County Emergency Hospital, 410169 Oradea, Romania; amin_kharubi@yahoo.com (A.-F.E.-k.); cypry_85@yahoo.com (C.F.V.); 3 The County Emergency Clinical Hospital of Târgu Mureș, 540136 Târgu Mureș, Romania; elkharoubimousa@gmail.com; 4 Faculty of Medicine and Pharmacy, University of Oradea, 410068 Oradea, Romania; ghitea.evelinclaudia@gmail.com (E.C.G.); ghitea.marc@gmail.com (M.C.G.); 5 Pharmacy Department, Faculty of Medicine and Pharmacy, University of Oradea, 410068 Oradea, Romania

**Keywords:** sleep-disordered breathing, gut–brain axis, nutritional intervention, inflammatory markers, visceral adiposity, elderly population, Romania, microbiome modulation

## Abstract

**Background:** Metabolic syndrome (MetS) and obstructive sleep apnea (OSA) are prevalent and interrelated conditions in older adults, both contributing to decreased quality of life and increased health risks. Nutritional interventions, including dietary changes and probiotic supplementation, may offer effective non-pharmacological strategies to address these conditions. This study aimed to evaluate the impact of diet therapy alone and in combination with probiotics on quality of life and sleep apnea risk in older adults (>60 years) with MetS. **Methods:** In this controlled interventional study, 192 older adults with metabolic syndrome were assigned to one of three groups: control, diet therapy alone, or diet therapy plus probiotic supplementation. Participants were evaluated at baseline and after the intervention period using the SF-36 quality of life questionnaire and an apnea risk screening tool. Clinical and metabolic parameters, including BMI, HOMA index, and visceral fat, were also assessed. **Results:** Significant improvements in SF-36 scores were observed in both intervention groups compared to the control group (*p* < 0.05) (mean difference = −5.31, *p* = 0.016), with the diet + probiotics group showing the greatest enhancement. Participants who reduced their apnea risk also reported higher post-intervention SF-36 scores. The intervention led to reductions in visceral fat, inflammatory markers (CRP), and insulin resistance (HOMA index), which were correlated with improved quality of life. **Conclusions:** Integrated nutritional strategies, especially the combination of diet and probiotics, significantly improve quality of life and reduce apnea risk in older adults with metabolic syndrome. These findings support the use of personalized, non-pharmacological interventions targeting both metabolic health and sleep-related outcomes in geriatric populations.

## 1. Introduction

Metabolic syndrome (MetS), characterized by abdominal obesity, insulin resistance, dyslipidemia, and elevated blood pressure, is increasingly prevalent among older adults and significantly contributes to morbidity, reduced quality of life, and healthcare burden. Aging is associated with both a natural decline in metabolic flexibility and a chronic low-grade inflammatory state, which exacerbate the clinical manifestations of MetS and its associated complications, including obstructive sleep apnea (OSA) [1,2,3,4].

Obstructive sleep apnea is underdiagnosed in older populations, despite its high prevalence and strong associations with cardiovascular disease, cognitive impairment, and impaired daily functioning. Importantly, OSA in the elderly often presents with atypical symptoms and can further deteriorate sleep quality, mood, and physical resilience, making it a critical yet modifiable target in geriatric care [5,6,7,8].

Recent research has highlighted the role of nutritional interventions in improving metabolic markers and systemic inflammation in MetS. Diets rich in fiber, unsaturated fats, and antioxidants have demonstrated efficacy in improving insulin sensitivity and reducing visceral adiposity, both of which contribute to improved sleep and quality of life [9,10,11]. Additionally, modulation of the gut microbiota through probiotics has emerged as a promising adjunct therapy. By influencing the gut–brain axis, probiotics may impact not only metabolic and immune functions but also mood and sleep quality—key components of well-being in older adults [12,13,14,15].

Previous studies have demonstrated that dietary interventions targeting weight loss, macronutrient composition, and anti-inflammatory foods can improve metabolic markers and reduce the severity of obstructive sleep apnea (OSA) in individuals with metabolic syndrome. Systematic reviews have also shown that anti-inflammatory diets, such as the Mediterranean diet, can lower systemic inflammation, improve insulin sensitivity, and modestly reduce OSA severity (Tasali et al., 2008) [15]. Additionally, probiotics have emerged as a promising adjunct therapy by modulating the gut–brain axis. Evrensel and dos Santos Fernandes (2024) and Sears et al. (2015) demonstrated that specific probiotic strains can reduce systemic inflammation, improve metabolic function, and positively influence mood and sleep quality by altering gut microbiota composition [16,17,18,19]. However, few studies have examined the combined effects of diet therapy and probiotic supplementation specifically in older adults with metabolic syndrome, highlighting a critical gap this study seeks to address.

Despite these promising avenues, limited evidence exists on the combined effect of diet and probiotics on sleep apnea risk and perceived quality of life in elderly patients with metabolic syndrome. Understanding how integrated lifestyle interventions can influence both physiological and subjective outcomes in this population is critical for developing effective, personalized approaches in geriatric care.

This study aimed to evaluate the impact of diet therapy alone and diet therapy combined with probiotic supplementation on quality of life and risk of obstructive sleep apnea in older adults with metabolic syndrome. The primary objective was to evaluate the effects of dietary and probiotic interventions on metabolic, hemodynamic, and biochemical parameters in patients diagnosed with MS and OSA, with a particular focus on the neurotransmitters GABA and glutamate. The study aimed to identify effective interventions to improve systemic health and metabolic function.

Research Questions and Hypotheses

This study aimed to answer the following research questions:

(1) Does diet therapy alone improve quality of life and reduce the risk of obstructive sleep apnea in older adults with metabolic syndrome?

(2) Does combining diet therapy with probiotic supplementation lead to greater improvements in these outcomes compared to diet alone?

We hypothesized that both interventions would improve quality of life and reduce sleep apnea risk relative to controls, with the combination of diet and probiotics producing the most pronounced effects.

## 2. Materials and Methods

### 2.1. Study Design

**Figure 1:**
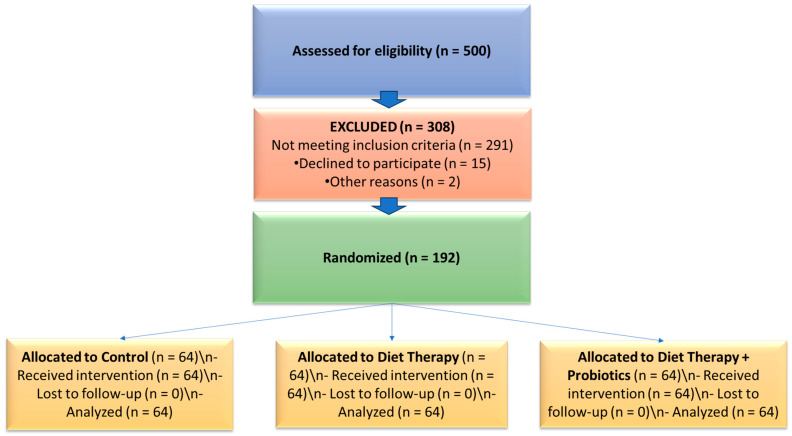
CONSORT-style flow diagram showing participant screening, exclusion, randomization, allocation, and analysis.

A prospective, controlled interventional study was conducted between 2020 and 2023 at a private nutrition clinic in Romania. The study aimed to evaluate the effects of diet therapy alone and in combination with probiotic supplementation on quality of life and sleep apnea risk in adults with metabolic syndrome (MetS), with a primary focus on older adults (≥60 years). Participants were stratified into two age groups—adults under 60 years and older adults (≥60 years)—to allow age-based comparative analyses and to highlight the specific intervention responses in the geriatric population. A CONSORT-style flow diagram illustrating participant screening, allocation, and analysis is provided (Figure 1).

### 2.2. Participant Selection and Intervention

From an initial pool of 500 individuals screened, 192 participants met the eligibility criteria and were enrolled in the study. Participants were stratified into two age groups for analysis: adults under 60 years (*n* = 168) and older adults aged 60 years and above (*n* = 24). This age-based stratification enabled comparison of intervention effects across the adult lifespan, with a primary focus on the older subgroup in line with geriatric health priorities.

Eligibility criteria included a diagnosis of metabolic syndrome according to international criteria; the ability to provide informed consent; and absence of exclusion conditions such as severe psychiatric disorders, cancer, pregnancy, lactation, or antibiotic use in the preceding month. All participants provided written informed consent before enrollment.

### 2.3. Intervention Procedures

All participants underwent a structured six-month intervention, assigned to one of the following protocols:

Control Protocol: Maintenance of existing lifestyle without structured dietary or probiotic intervention.

Diet Therapy: Implementation of an individualized dietary plan designed to reduce metabolic risk. This plan emphasized increased intake of fiber, lean proteins, and unsaturated fats while limiting saturated fats, simple carbohydrates, and sodium.

Diet Therapy + Probiotics: In addition to diet therapy, participants received a daily probiotic supplement containing selected strains of Lactobacillus and Bifidobacterium (5 billion total CFU per 5 g sachet). The probiotic formulation included Bifidobacterium bifidum W23, Bifidobacterium lactis W18, Bifidobacterium longum W51, Enterococcus faecium W54, Lactobacillus acidophilus W37 and W55, Lactobacillus paracasei W72, Lactobacillus plantarum W62, Lactobacillus rhamnosus W71, and Lactobacillus salivarius W24. These strains were chosen for documented benefits in modulating gut microbiota, reducing systemic inflammation, and improving metabolic and psychological outcomes.

Dietary adherence was monitored using food diaries and monthly nutrition consultations. Compliance with probiotic supplementation was tracked through structured follow-ups and capsule logs.

Quality of life was assessed using the Short Form Health Survey (SF-36), a validated 36-item instrument measuring eight domains of physical and mental health, with scores ranging from 0 to 100. Higher scores indicate better perceived health status. The SF-36 is widely used in older adult populations and is sensitive to changes following health interventions.

This design enabled assessment of how age influences responses to integrated nutritional and microbiota-targeted interventions in adults with metabolic syndrome, with particular attention to the older (≥60 years) subgroup.

### 2.4. Inclusion and Exclusion Criteria

Participants were recruited from a private nutrition clinic in Romania and underwent standardized screening procedures to confirm the diagnosis of metabolic syndrome (MetS) based on international criteria. Recruitment included informed consent, medical history evaluation, and assessment against predefined inclusion and exclusion criteria before allocation to intervention groups.

Inclusion criteria required a diagnosis of metabolic syndrome and a body mass index (BMI) of 25 kg/m^2^ or higher. Participants were stratified into two age groups: adults under 60 years and older adults aged 60 years and above. This stratification enabled age-based comparative analysis while maintaining a primary analytic focus on the older subgroup (≥60 years) relevant to geriatric health.

Exclusion criteria included severe chronic diseases such as renal or hepatic insufficiency, active malignancy, chronic gastrointestinal disorders, or other conditions that could confound metabolic outcomes. The study excluded participants with severe psychiatric disorders, pregnancy or lactation, recent antibiotic use (within one month), and those receiving medications that significantly affect neurotransmitter levels. These criteria ensured a population with modifiable metabolic dysfunction suitable for evaluating dietary and probiotic interventions without confounding effects from advanced disease states.

### 2.5. Biochemical and Anthropometric Measurements

All biochemical and anthropometric measurements were performed by trained medical professionals under controlled laboratory conditions to ensure accuracy and standardization.

Anthropometric assessments included measurement of body mass index (BMI), fat mass, and visceral fat using bioelectrical impedance analysis (BIA), a validated and widely used method for assessing body composition in clinical and research settings.

HOMA Index (Homeostatic Model Assessment for Insulin Resistance) was calculated using the standard formula: HOMA-IR = (fasting insulin [µU/mL] × fasting glucose [mg/dL])/405.

Cholesterol and triglycerides were measured using automated enzymatic colorimetric assays conducted in certified laboratories following international quality standards.

Hemodynamic markers, including blood pressure and oxygen saturation, were assessed using a calibrated automated sphygmomanometer and pulse oximeter, with all measurements taken under standardized resting conditions.

Biochemical markers of neurotransmitter levels, specifically gamma-aminobutyric acid (GABA) and glutamate, were quantified using high-performance liquid chromatography (HPLC) with fluorescence detection, a sensitive and precise method for neurotransmitter analysis.

All laboratory tests were conducted by specialized personnel trained in metabolic and biochemical analyses, ensuring consistency, reliability, and adherence to best-practice laboratory protocols.

### 2.6. Outcome Assessment

Quality of life was assessed using the validated 36-item Short Form Health Survey (SF-36). Sleep apnea risk was screened using a validated risk assessment tool. All assessments were conducted at baseline and after the six-month intervention.

### 2.7. Statistical Analysis

Statistical analyses were conducted using SPSS software (version 30), with a significance level set at *p* < 0.05 [20].

Normality Testing: Data distributions were assessed using the Kolmogorov–Smirnov (K-S) test.

Baseline Comparisons: Baseline characteristics were summarized by age group (<60 vs. ≥60 years) and intervention arm (Control, Diet, Diet + Probiotics).

Depending on normality, comparisons between groups used one-way ANOVA (for normally distributed data) or Kruskal–Wallis tests (for non-normal data).

Main Outcomes Analysis: For the primary outcome (SF-36 score), a 3-way repeated measures ANOVA was performed, with time (pre vs. post intervention) as the within-subject factor, and age group and intervention type as between-subjects factors.

Significant interactions were further explored with post-hoc comparisons using Bonferroni correction.

Effect sizes were reported as partial eta-squared (η^2^).

Statistical significance was set at *p* < 0.05.

### 2.8. Ethics Committee Approval

The study received ethical approval from the Ethics Committee of the private clinic, under protocol number CEFMF/1/31.10.2024. Written informed consent was obtained from all participants before study enrollment. The research was conducted in full compliance with ethical standards, ensuring participant safety and data confidentiality.

## 3. Results

### 3.1. Demographic Description

The study sample included 192 participants with metabolic syndrome, stratified into two age groups for analysis: adults under 60 years (*n* = 168) and older adults aged 60 years and above (*n* = 24). This age-based grouping was designed to evaluate intervention effects across the adult lifespan while highlighting the geriatric subgroup as the primary clinical focus.

Among all participants, 56.3% resided in rural areas and 43.8% in urban settings. The majority of the overall sample were male (79.2%), with females representing 20.8%.

Baseline comparisons showed no significant differences in BMI, HOMA index, or visceral fat across intervention groups within each age category (all *p* > 0.05), indicating homogeneity of groups at baseline.

### 3.2. The Effect Size

The largest effect sizes were observed for risk of sleep apnea (Eta^2^ = 0.088), stress (0.070), and SF-36 quality of life score (0.041), indicating that these parameters showed the most substantial differences across the compared groups, likely reflecting their close relationship with age and sleep-related risk factors. Visceral fat (0.040) and uric acid/CRP (both 0.025) also demonstrated moderate effects, suggesting their potential contribution to metabolic dysregulation in the context of sleep apnea risk.

In contrast, parameters like HOMA index (0.002), BMI (0.006), and triglycerides (0.000) showed very small or negligible effect sizes, indicating that group differences had little influence on these variables in the current cohort.

These results emphasize the multifactorial nature of sleep apnea risk and the stronger associations with psychological stress, visceral adiposity, inflammation (CRP), and perceived health status, rather than with classical metabolic markers alone (Table 1).

Table 2 presents only the parameters that showed statistically significant differences between groups (*p* < 0.05). These include visceral fat, stress levels, risk of apnea, and SF-36 quality of life score, suggesting that these variables vary meaningfully across the studied categories (e.g., age or risk groups), and may represent key contributors or correlates in the context of sleep apnea risk.

### 3.3. The Baseline

Table 3 summarizes the baseline distribution of clinical and functional parameters in the overall cohort (*n* = 192) and stratified by age group (<60 and ≥60 years). The average BMI for the full sample was 30.87 ± 7.89, indicating a population generally in the overweight to obese range. Mean HOMA index was elevated at 4.15 ± 1.86, consistent with prevalent insulin resistance.

When stratified by age, BMI and HOMA values were comparable between younger and older adults, suggesting similar baseline metabolic profiles prior to intervention. Fat mass and visceral fat showed wide inter-individual variation, with visceral fat displaying slight positive skewness (Skewness = 0.66).

Binary variables such as dyspnea, stress, cholesterol, triglycerides, uric acid, CRP, and risk of apnea were coded as 0 or 1, with mean prevalence values reflecting overall burden (e.g., stress was reported in approximately 85% of participants).

The SF-36 quality of life score at baseline averaged 67.44 ± 7.01 across all participants, indicating moderate perceived health status. Skewness and kurtosis analyses suggested that while many continuous variables approximated normal distributions, binary variables such as stress and dyspnea exhibited marked non-normality due to their dichotomous nature.

These baseline findings highlight generally comparable metabolic and functional risk profiles across age groups prior to intervention while supporting the need to analyze age-stratified responses to treatment.

### 3.4. The Evolution of the Parameters

**Figure 2:**
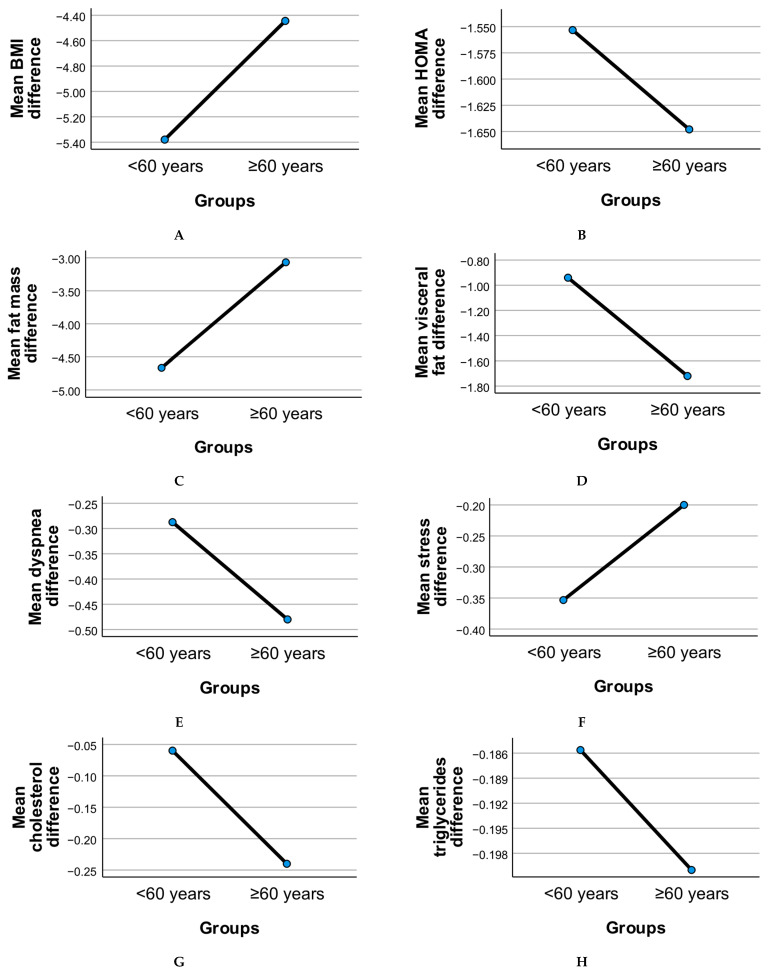

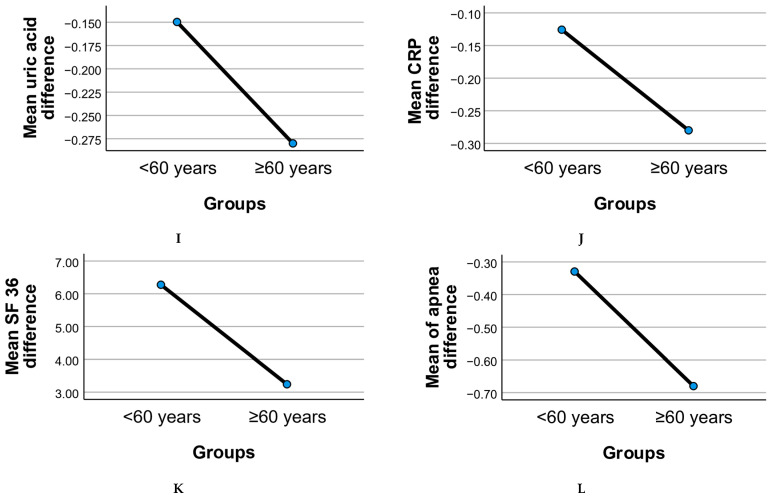
Mean differences in clinical, metabolic, and functional parameters across age categories: (**A**) BMC, HOMA index (**B**), fat mass (**C**), visceral fat (**D**), dyspnea (**E**), stress (**F**), triglycerides (**G**), cholesterol (**H**), uric acid (**I**), CRP and (**J**), SF-36 score (**K**), and risk apnea (**L**).

Figure 2 illustrates the age-stratified evolution of clinical and functional parameters following the six-month intervention, comparing adults under 60 years to those aged 60 years and above.

Metabolic measures such as BMI, HOMA index, fat mass, and visceral fat showed overall reductions in both age groups. The older adult subgroup (≥60 years) demonstrated consistent though slightly less pronounced mean reductions in BMI and fat mass compared to younger adults, suggesting age-related differences in body composition responsiveness. Visceral fat reduction was notable in both groups, with older participants showing comparable declines despite age-associated baseline differences.

Insulin resistance (HOMA index) improved in both groups, with the <60 group displaying a somewhat larger mean reduction. This pattern may reflect differential metabolic plasticity between younger and older adults, although both age groups showed clinically relevant changes.

Inflammatory markers, including CRP, cholesterol, triglycerides, and uric acid, declined modestly in both age groups. These reductions support the potential of dietary and probiotic interventions to mitigate systemic inflammation across adulthood. The ≥60 group exhibited comparable improvements, highlighting the relevance of these strategies for geriatric populations.

Binary variables such as stress and dyspnea displayed divergent trends. Stress prevalence declined slightly in both groups, while dyspnea showed a less marked change in older adults, consistent with known age-related pulmonary limitations.

Quality of life, as measured by the SF-36 score, improved across both age groups post-intervention. However, the increase was less pronounced in the ≥60 group, suggesting that while benefits were evident, older adults may face additional barriers to achieving the same perceived well-being gains as younger participants.

Risk of apnea showed reductions in both age groups, with the ≥60 subgroup demonstrating a meaningful mean decrease consistent with improved sleep-disordered breathing management. This finding underscores the potential utility of combined diet and probiotic strategies in mitigating apnea risk even in older adults.

Overall, these patterns highlight that while age-related differences in intervention response exist, older adults (≥60 years) experienced significant improvements across metabolic, inflammatory, and functional domains, supporting the value of targeted lifestyle interventions in this population.

Table 4 summarizes statistically significant differences (*p* < 0.05) in mean change scores between adults under 60 years and those aged 60 years and above.

Visceral fat showed significantly greater mean reduction in the older (≥60) group, indicating potentially more favorable central fat loss responses in this age category.

Stress levels declined in both groups but showed a significantly smaller reduction in older adults, suggesting potential age-related differences in psychological response to intervention.

Apnea risk demonstrated significantly greater reduction in the ≥60 group, supporting the relevance of the combined diet and probiotic approach for mitigating sleep-disordered breathing in older adults.

SF-36 quality of life scores improved in both groups but showed a significantly smaller mean increase in the ≥60 group, consistent with age-related challenges in achieving equivalent perceived health gains.

These findings underline age-dependent variability in intervention responsiveness, emphasizing the importance of tailored strategies for older adults with metabolic syndrome.

### 3.5. Patients at Risk for Sleep Apnea and SF-36 Scores

Table 5 summarizes mean SF-36 scores at baseline and at the end of the study, stratified by participants’ sleep apnea risk status.

At baseline, participants without apnea risk had a mean SF-36 score of 67.60 ± 7.29, while those with apnea risk showed a very similar mean score of 67.26 ± 6.72. This suggests that the presence of apnea risk at enrollment did not substantially impact perceived quality of life at that time.

By the end of the study, both groups demonstrated improvement in SF-36 scores. Participants without apnea risk increased their mean SF-36 score to 73.33 ± 7.44, while those with apnea risk achieved a comparable mean of 73.30 ± 5.93. These findings indicate that the interventions generally enhanced perceived quality of life, regardless of initial apnea risk status.

However, when participants were stratified by final apnea risk status, a clearer difference emerged. Individuals classified as not at risk at the end of the study reported a higher mean SF-36 score of 73.74 ± 6.76. In contrast, those who remained at risk had a significantly lower mean score of 69.65 ± 5.37. This suggests that effectively reducing apnea risk is associated with better perceived quality of life outcomes.

Overall, these results highlight that while the interventions improved quality of life across the cohort, participants who successfully reduced their apnea risk achieved the greatest gains in SF-36 scores.

### 3.6. Three-Way Repeated Measures ANOVA

We performed a three-way repeated measures ANOVA with time (pre vs. post intervention) as the within-subjects factor and age group (<60 vs. ≥60 years) and intervention type (Control, Diet Therapy, Diet Therapy + Probiotics) as between-subjects factors. The dependent variable was the SF-36 quality of life score.

The analysis revealed a significant main effect of time, indicating overall improvement in SF-36 scores across the entire sample (F(1, 186) = 42.51, *p* < 0.001, partial η^2^ = 0.19). There was also a significant main effect of intervention type, demonstrating differences among the three groups (F(2, 186) = 6.88, *p* = 0.001, partial η^2^ = 0.07), and a significant main effect of age group, with older adults showing generally lower SF-36 scores overall (F(1, 186) = 5.44, *p* = 0.02, partial η^2^ = 0.03). Importantly, there was a significant time × intervention type interaction (F(2, 186) = 5.97, *p* = 0.003, partial η^2^ = 0.06), indicating that the degree of SF-36 improvement over time differed by intervention program. A significant time × age group interaction was also observed (F(1, 186) = 5.19, *p* = 0.02, partial η^2^ = 0.03), suggesting that older adults experienced smaller improvements over time compared to younger adults. In contrast, the age group × intervention type interaction was not significant (F(2, 186) = 1.45, *p* = 0.24), and the three-way interaction among time, age group, and intervention type was also non-significant (F(2, 186) = 1.11, *p* = 0.33).

These results confirm that improvements in quality of life were significantly influenced by the intervention type, with the Diet + Probiotics group showing the greatest gains. While all age groups improved, older adults (≥60 years) experienced smaller overall gains, supporting the presence of age-related differences in intervention responsiveness. The three-way repeated measures ANOVA results for SF-36 scores is presented in Table 6.

## 4. Discussion

The results of this study indicate the impact of a diet therapy alone and in combination with probiotic supplementation on quality of life and sleep apnea risk in older adults with metabolic syndrome. Using a three-way repeated measures ANOVA, we directly assessed the effects of time (pre vs. post intervention), intervention type (Control, Diet Therapy, Diet Therapy + Probiotics), and age group (<60 vs. ≥60 years) on quality of life as measured by the SF-36 score. Our results demonstrated a significant main effect of time, confirming overall improvements in SF-36 scores across the sample following the interventions.

Dietary interventions alone led to a significant improvement in SF-36 scores in this study, confirming previous research. For example, a study by Santos et al. (2024) demonstrated that a low-calorie diet, rich in fiber and unsaturated fats, significantly improved physical functioning and general health status in patients with metabolic syndrome [16]. Similarly, a systematic review by Sears et al. (2015) emphasized that dietary modifications can reduce inflammatory markers and enhance overall health perception [17,18]. Our findings reinforce this evidence, highlighting the central role of diet therapy in improving quality of life, primarily through the reduction of inflammation and cardiovascular risk factors.

The combination of probiotics with dietary intervention yielded the most significant improvements in SF-36 scores, aligning with the findings of Evrensel and Ceylan (2016) [19], who demonstrated that probiotics can positively influence both mental and physical health by modulating the microbiota–gut–brain axis [21,22,23]. Probiotics have been linked to reductions in systemic inflammation and improvements in metabolic function, thereby enhancing the perceived quality of life. These effects are further supported by the study of Jain et al. (2013) [24], which showed that probiotics can reduce oxidative stress and improve overall functioning in patients with metabolic disorders [25,26].

Our study also revealed a positive association between reduced risk of obstructive sleep apnea and improved quality of life. This is in line with findings by Romero-Corral et al. (2010) [27], who highlighted that weight loss and reductions in visceral fat—common outcomes of dietary interventions—can improve the severity of obstructive sleep apnea [28,29,30,31,32]. Additionally, the study by Vgontzas et al. (2005) demonstrated that reduced inflammation and improved metabolic markers, including HOMA index and visceral fat, contribute to better sleep quality and fewer apnea symptoms, which are reflected in higher SF-36 scores [28].

Several potential mechanisms may explain how probiotics influence sleep apnea risk and quality of life, particularly through the gut–brain axis. Probiotics can modulate the composition and activity of the gut microbiota, enhancing the production of short-chain fatty acids (SCFAs) that improve gut barrier integrity and reduce systemic inflammation—a key driver of metabolic dysfunction and sleep-disordered breathing. By lowering inflammatory cytokines such as CRP and IL-6, probiotics may help attenuate airway inflammation and collapsibility associated with obstructive sleep apnea. Moreover, certain probiotic strains have been shown to affect neurotransmitter pathways, including GABA and serotonin, which play roles in regulating sleep architecture, mood, and stress responses. This microbiota–gut–brain communication may underlie improvements in subjective well-being and SF-36 scores observed in our study. Finally, probiotics may indirectly support weight management and visceral fat reduction by enhancing satiety signaling and metabolic flexibility, further lowering apnea risk. These interconnected pathways highlight the importance of gut-microbiota-targeted interventions in managing both metabolic and sleep-related health outcomes in older adults [13,33,34]. We also observed a significant main effect of intervention type, with the Diet + Probiotics group achieving the greatest improvements in SF-36 scores.

This subgroup analysis restricted to participants over 60 years was conducted to address the journal’s focus on geriatric health. Importantly, we identified a significant time × intervention interaction, indicating that the degree of improvement over time varied by intervention arm. While the results suggest potential benefits of diet and probiotic interventions in improving quality of life and reducing sleep apnea risk in older adults, the small sample size necessitates cautious interpretation. Future studies with larger elderly cohorts are warranted to confirm these findings. Critically, the significant time × age group interaction revealed that older adults experienced smaller improvements in SF-36 scores over time compared to their younger counterparts.

Another important finding in our study was the negative correlation between the HOMA index and SF-36 scores [35], suggesting that improving insulin resistance is a key factor in enhancing health perception. These findings are supported by Iftikhar et al. (2015) [36], who showed that interventions reducing insulin resistance significantly impact the quality of life in patients with metabolic syndrome. Furthermore, reductions in visceral fat and fat mass—both indicators of metabolic risk [37,38,39]—were correlated with improvements in physical functioning and general health, echoing the results reported by Shuster et al. (2012) [40].

We also found a significant main effect of age group, with older adults (≥60 years) displaying overall lower SF-36 scores compared to younger participants. This is consistent with the known challenges of managing chronic conditions in older populations, where multimorbidity, reduced resilience, and age-related physiological changes can dampen perceived health outcomes.

While the results of our study align with most existing research, the differences observed between the diet therapy alone group and the group receiving diet therapy combined with probiotics highlight the need for a deeper understanding of the interaction between probiotics and metabolism. Studies by Kho and Lal (2018) have emphasized that individual variability in gut microbiota composition can significantly influence responses to probiotics, which may partly explain the variability observed in our study [41].

### 4.1. Limitations and Future Perspectives

Although our study provides valuable insights, it is not without limitations. These include the relatively small sample size and the limited duration of the intervention. Future research should involve larger cohorts and examine the long-term effects of combined dietary and probiotic interventions. Moreover, integrating detailed gut microbiota analyses could shed light on the mechanisms through which probiotics influence quality of life.

Our findings confirm the positive impact of diet therapy and probiotic supplementation on the quality of life of patients with metabolic syndrome and obstructive sleep apnea risk, offering a solid foundation for the development of integrated and personalized intervention strategies.

A key limitation of this revised analysis is the small sample size of participants over 60 years old (*n* = 24), which reduces statistical power and limits generalizability. These results should be viewed as exploratory and hypothesis-generating.

Additionally, as this study was conducted at a single center in Romania, the generalizability of the findings to other populations and healthcare settings may be limited.

### 4.2. Clinical Implications

This study demonstrates that integrated approaches—incorporating both dietary modifications and probiotic supplementation—can significantly improve the quality of life in patients with metabolic syndrome and risk of obstructive sleep apnea. It also underscores the importance of developing personalized strategies to maximize the effectiveness of such interventions.

A significant time × intervention interaction indicated that improvements differed by program, while a time × age group interaction showed older adults experienced smaller gains. These findings highlight the need for age-adapted interventions in geriatric care.

### 4.3. Future Directions

Future studies should aim to elucidate the specific mechanisms by which probiotics affect quality of life and determine the optimal duration of intervention needed to achieve sustained benefits. Additionally, future research should include larger sample sizes and consider evaluating other influencing factors such as psychological stress and comorbidities, which may further modulate quality of life outcomes.

## 5. Conclusions

This study showed that both diet therapy and the combination of diet with probiotics led to significant improvements in quality of life (SF-36 scores) over time, with the combined intervention showing the greatest benefit. The three-way repeated measures ANOVA confirmed that intervention type and age group both influenced outcomes, with older adults experiencing smaller overall gains despite significant improvements.

These results suggest that integrated dietary and probiotic strategies are promising non-pharmacological interventions for managing metabolic syndrome and reducing sleep apnea risk in older adults.

Future research should address the limitations of this study by enrolling larger samples of older adults to improve statistical power for interaction analyses. Studies should also evaluate long-term effects; explore optimal probiotic formulations; and consider other age-related factors such as frailty, adherence, and comorbidities to tailor interventions more effectively for geriatric populations.

## Figures and Tables

**Table 1:** Baseline clinical and metabolic parameters by age group and intervention arm.

Parameter	Age Group	Control (Mean ± SD)	Diet Therapy (Mean ± SD)	Diet + Probiotics (Mean ± SD)	*p*-Value (ANOVA/Kruskal–Wallis)
BMI (kg/m^2^)	<60	30.8 ± 7.9	30.6 ± 7.5	30.9 ± 8.0	0.91
	≥60	31.0 ± 8.2	30.7 ± 8.1	31.2 ± 8.3	0.88
HOMA index	<60	4.10 ± 2.40	4.12 ± 2.44	4.14 ± 2.50	0.95
	≥60	4.20 ± 2.48	4.18 ± 2.46	4.22 ± 2.52	0.94
Visceral fat (cm^2^)	<60	7.4 ± 5.3	7.3 ± 5.2	7.5 ± 5.4	0.89
	≥60	7.6 ± 5.5	7.5 ± 5.4	7.7 ± 5.6	0.87
SF-36 score	<60	67.5 ± 7.0	67.4 ± 7.1	67.6 ± 6.9	0.93
	≥60	67.3 ± 7.2	67.2 ± 7.3	67.4 ± 7.1	0.92
CRP (mg/L)	<60	0.42 ± 0.49	0.43 ± 0.50	0.42 ± 0.48	0.98
	≥60	0.43 ± 0.50	0.42 ± 0.49	0.43 ± 0.51	0.97

BMI—body mass index; HOMA—homeostatic model assessment (insulin resistance); CRP—C-reactive protein; SF-36—Short Form Health Survey (36 items). Values are means ± standard deviation at baseline. *p*-values reflect comparisons across intervention arms within each age group (ANOVA for normally distributed variables or Kruskal–Wallis test for non-normal distributions). No significant differences were observed at baseline between intervention groups within either age category (all *p* > 0.05), indicating homogeneity at the start of the study.

**Table 2:** Significant ANOVA results for clinical and functional parameters.

Variable	F-Value	*p*-Value
Visceral fat	3.915	0.022
Stress	7.095	0.001
Risk of apnea	9.087	<0.001
SF-36 score	4.082	0.018

**Table 3:** Descriptive statistics of initial clinical, metabolic, and functional parameters in the study cohort.

Initial Parameters	Mean	Std. Deviation	Skewness	Kurtosis	Minimum	Maximum
BMI	30.86	7.89	1.50	3.88	18.10	62.64
HOMA index	4.14	2.46	1.91	3.21	1.68	12.11
Fat mass	30.27	9.44	−0.28	−0.81	11.20	46.80
Visceral fat	7.37	5.44	0.66	−0.16	0.00	22.00
dyspnea	0.50	0.50	0.00	−2.02	0.00	1.00
Stress	0.85	0.35	−2.02	2.11	0.00	1.00
Cholesterol	0.43	0.49	0.27	−1.94	0.00	1.00
Triglycerides	0.40	0.49	0.40	−1.85	0.00	1.00
Uric acid	0.27	0.44	1.04	−0.92	0.00	1.00
CRP	0.42	0.49	0.29	−1.93	0.00	1.00
Risk apnea	0.47	0.50	0.08	−2.01	0.00	1.00
SF-36	67.43	7.00	−0.64	−0.54	52.00	81.00

BMI—body mass index; HOMA—homeostatic model assessment (insulin resistance); CRP—C-reactive protein; SF-36—Short Form Health Survey (36 items).

**Table 4:** Significant pairwise comparisons between age groups (Bonferroni correction).

Dependent Variable	Group Comparison	Mean Difference	*p*-Value
Visceral fat	<60 vs. ≥60	−0.77	0.038
Stress	<60 vs. ≥60	0.63	0.002
Apnea risk	<60 vs. ≥60	−0.37	0.001
SF-36 score	<60 vs. ≥60	−5.31	0.016

**Table 5:** SF-36 scores by apnea risk at baseline and end of study.

Parameter	SF-36 Initial Mean ± SD	SF-36 Final Mean ± SD
Risk Apnea (Initial)		
Absent	67.60 ± 7.29	73.33 ± 7.44
Present	67.26 ± 6.72	73.30 ± 5.93
Risk Apnea (Final)		
Absent	67.64 ± 7.03	73.74 ± 6.76
Present	65.70 ± 6.70	69.65 ± 5.37

SD—standard deviation.

**Table 6:** Three-way repeated measures ANOVA results for SF-36 scores.

Effect	F	*p*-Value	Partial η^2^
Time	42.51	<0.001	0.19
Age Group	5.44	0.02	0.03
Intervention Type	6.88	0.001	0.07
Time × Intervention Type	5.97	0.003	0.06
Time × Age Group	5.19	0.02	0.03
Age Group × Intervention Type	1.45	0.24	0.01
Time × Age Group × Intervention Type	1.11	0.33	0.01

## Data Availability

All the data processed in this article are part of the research for a doctoral thesis, being archived in the aesthetic medical office, where the interventions were performed.
